# Targeting memory T cell metabolism to improve immunity

**DOI:** 10.1172/JCI148546

**Published:** 2022-01-04

**Authors:** Mauro Corrado, Erika L. Pearce

**Affiliations:** 1 Cologne Excellence Cluster on Cellular Stress Responses in Aging-Associated Diseases (CECAD), Cologne, Germany.; 2 Department of Oncology, The Bloomberg~Kimmel Institute for Cancer Immunotherapy, Johns Hopkins University, Baltimore, Maryland, USA.

## Abstract

Vaccination affords protection from disease by activating pathogen-specific immune cells and facilitating the development of persistent immunologic memory toward the vaccine-specific pathogen. Current vaccine regimens are often based on the efficiency of the acute immune response, and not necessarily on the generation of memory cells, in part because the mechanisms underlying the development of efficient immune memory remain incompletely understood. This Review describes recent advances in defining memory T cell metabolism and how metabolism of these cells might be altered in patients affected by mitochondrial diseases or metabolic syndrome, who show higher susceptibility to recurrent infections and higher rates of vaccine failure. It discusses how this new understanding could add to the way we think about immunologic memory, vaccine development, and cancer immunotherapy.

## Introduction

Immunologic memory is the primary goal of vaccination. This phenomenon is characterized by qualitatively and quantitatively improved and/or enhanced antigen/epitope-specific recognition by B and T cells of the adaptive immune system (1). B and T cells have different but cooperative roles in responding to infections. Memory B cells generate high-affinity neutralizing antibodies that, when produced at sufficient levels, can prevent viruses and bacteria from infecting cells. Memory T cells (Tm cells) also participate in this protection, as their rapid expansion and cytotoxic properties facilitate pathogen control and clearance, thus limiting or ablating pathology development (2).

Even though the generation of durable and persistent immunologic memory is the basis of any successful vaccination, the mechanisms underlying induction and maintenance of immunologic memory remain elusive. In particular, it is still debated exactly how Tm cells are generated upon acute infection, how such long-lived cells are induced and differentiate from effector T (Teff) cells, and which mechanisms control their survival and enhanced function for years if not decades (3). In this context, many vaccines mainly elicit B cell responses, poorly priming T cells. This is often the case with subunit vaccines whose antigens are able to elicit a strong B cell response, but for which antigen processing and presentation are inadequate to properly activate T cells, which require antigen recognition in the context of major histocompatibility complex (MHC) on antigen-presenting cells (APCs) (4, 5). Thus, clarifying our understanding of how Tm cells are generated and persist long-term might be a critical step in the development of efficient T cell–targeted vaccines.

How lymphocytes develop effector and memory phenotypes has been attributed to cell-intrinsic mechanisms involving prolonged cellular longevity (6, 7), posttranslational regulation of key proteins (8), and epigenetic reprogramming of the cellular transcriptome (9), or to cell-extrinsic mechanisms linked to antigen presentation and costimulatory signals (10–12). In recent years, the emerging field of immunometabolism has started to unveil the role of metabolism in shaping immune function, and to reveal how modulating cell or organismal metabolism can affect immune cell differentiation (13).

## The dynamic nature of mitochondria in T cells

Cells constantly sense nutrient availability in their microenvironment, adapting function and survival to metabolic state. Driving this adaptation, mitochondria fine-tune their function in response to the dynamic metabolic requirements of the cell (14). Mitochondrial biogenesis is triggered in response to higher metabolic needs, while selective autophagy removes dysfunctional organelles (15, 16). Changes in mitochondrial morphology couple location and shape of mitochondria to efficient energy production (17). Mitochondria fuse and divide, forming interconnected networks of filamentous organelles or isolated fragmented units (18). Mitochondrial ultrastructure also varies greatly, so that cells with highly efficient oxidative phosphorylation (OXPHOS) have mitochondria with tight cristae (invaginations of inner mitochondrial membrane) that are associated with higher supramolecular organization of respiratory chain complexes in supercomplexes (19). Furthermore, mitochondria are central signaling hubs, computing complex signaling networks and communicating with the nucleus (20). This extraordinary mitochondrial plasticity is critical to T cells, which constantly surveil their environment, patrolling tissues and trafficking to and from lymphoid organs (13).

**Figure 1:**
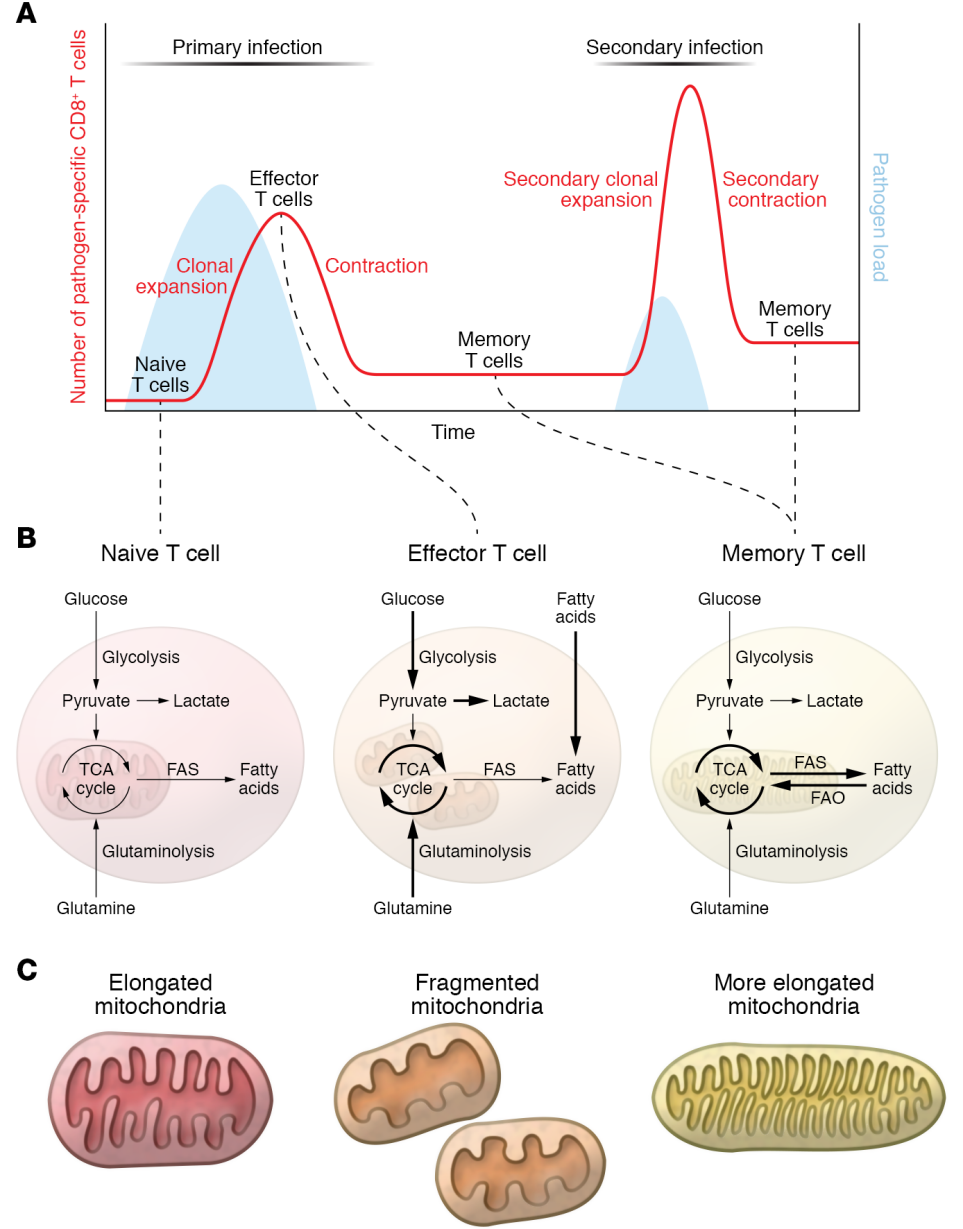
Metabolic pathways in naive, effector, and memory T cells. (**A**) Schematic of the dynamic of T cell immune response upon primary and secondary infection, depicting T cell differentiation from Tn, to Teff, and Tm cells. (**B**) Metabolic features of naive, effector, and memory T cells. Briefly, naive T cells are metabolically quiescent, relying on basal levels of OXPHOS for their energetic needs. Upon activation, effector T cells become highly metabolically active, increasing their substrate uptake together with glycolysis and OXPHOS. During memory T cell differentiation, metabolism rewires to a more quiescent state in which FAO and OXPHOS sustain T cell survival and energetic requirements. (**C**) Illustrations of the different mitochondrial morphology and ultrastructure observed in T cell subtypes. Mouse Tn cells and in vitro–differentiated IL-15 Tm cells show elongated mitochondria, while in vitro–differentiated IL-2 Teff cells display fragmented mitochondria. IL-2 Teff cells show wider cristae structure compared with the tight and elongated structure observed in IL-15 Tm cells. FAS, fatty acid synthesis.

T cells coordinate multiple aspects of adaptive immunity, including responses to pathogens, allergens, and tumors. While doing so, they modulate metabolism depending on antigen-driven and microenvironmental signals (Figure 1, A and B) (13). Upon acute infection, naive T (Tn) cells activate and expand vigorously, generating Teff cells able to recruit other immune cells and directly kill pathogen-infected cells. Once the infection is cleared, Teff cells are no longer necessary and undergo contraction to avoid excess tissue destruction. However, not all T cells specific for a pathogen die, as a small population of Tm cells persist and are responsible for the long-term immune memory and protection (21). The dynamic nature of T cells is also reflected by changes in their metabolic state during an immune response. Briefly, Tn cells are metabolically quiescent, mainly relying on OXPHOS for their energetic needs and survival. In contrast, Teff cells are metabolically very active, with higher rates of glycolysis and OXPHOS coupled to their highly proliferative state (22). Tm cells rewire metabolism toward a more quiescent, but metabolically primed, state, relying mainly on catabolic processes such as fatty acid oxidation (FAO) and OXPHOS (23). A common feature of Tm cells is the ability to respond more quickly and more strongly to a previously encountered antigen, which results in limited or no development of pathology and rapid control of infection (24). Immune recognition and control of recurrent cancers can be less efficient than what develops as a result of infection, as some tumors undergo mutational changes that alter cellular epitopes, express inhibitory receptors, or generate an immunosuppressive tumor microenvironment that dampens the immune response (25).

## Early signals for a distant fate

T cell activation, mitochondrial morphology, and autophagy each play a role in Tm cell development. Upon activation with cognate antigen and costimulatory signals in an inflammatory cytokine milieu, CD8^+^ Tn cells undergo extensive clonal expansion and differentiation to generate T lymphocytes with cytotoxic properties (CTLs). This large pool of CTL Teff cells contains two distinct subsets of short-lived effector cells (SLECs) and memory precursor effector cells (MPECs). SLECs rapidly die after pathogen clearance, while MPECs are characterized by long-term survival. The balance between SLECs and MPECs can be modulated by duration of antigen stimulation, cytokine, and costimulatory signals (26–30). During activation, T cell receptor signaling without appropriate costimulation elicits primary Teff cells, but fails to generate competent Tm cells (31). The interaction between the T cell coreceptor CD28 and the B7 molecules CD80 and CD86 on activated APCs prevents T cell anergy and allows development of Tm cells by regulating the cell cycle, cytokine production, and the epigenetic and transcriptional landscape (11, 32). Metabolically, CD28 was originally characterized to promote higher and more efficient glycolytic flux during activation via engagement of PI3K and Akt, which in turn upregulate mammalian target of rapamycin (mTOR) activity (33, 34). Our group showed that CD28 costimulation during T cell activation provides important signals to the mitochondria, transiently promoting the early expression of carnitine palmitoyltransferase 1a (CPT1a) before the first cell division and, thus, promoting FAO. Further, CD28 signals restrain mitochondrial cristae loosening and endow cells with enhanced spare respiratory capacity after primary and secondary activation (30), allowing the generation of competent Tm cells. Other groups showed a similar function for 4-1BB costimulation (35). From an immunotherapy perspective it is of note that the failure of first-generation chimeric antigen receptor (CAR) T cells in efficiently priming resting T cells and generating long-lasting Tm cells was overcome by the engineering of constructs harboring costimulatory signaling domains (e.g., CD28 or 4-1BB) together with the CD3ζ domain (36–40). Moreover, it has been appreciated that the 4-1BB domain is better for the generation of long-lived cells compared with CD28 (41–43), with 4-1BB favoring a mitochondrial metabolic signature (44). Thus, costimulatory molecules present during activation are able to modulate metabolism and function of Tm cells long after activation signals are gone. Understanding the exact mechanisms underlying this phenomenon may be critical to improving immunotherapy or efficient T cell priming upon vaccination.

Cytokine milieu also plays a central role during activation. Duration and strength of IL-2 signals control the extent of T cell proliferation, as well as the pool of Tm cells generated (45–50). IL-2 also supports the proliferative capacity of T cells by modulating T cell metabolism as it stimulates the expression of the transcription factor Myc, the activation of mTOR complex 1 (mTORC1), and the stabilization of hypoxia-inducible factor 1α (HIF1α) to support the uptake of nutrients such as amino acids and glucose that are necessary to rapidly synthesize nucleotides, lipids, and proteins (51). Asymmetric cell division (and thus asymmetric activation of mTOR, Myc, and PI3K; refs. 52–55) at the first round of division after activation is also able to dictate future effector/memory phenotypes, with the daughter cell distal to the APC having an increased propensity to acquire a memory phenotype, and the proximal cell to the APC more prone to be a SLEC (56). Moreover, during the effector phase, T cells with lower levels of glycolysis, Akt, or mTOR activation, and mitochondrial membrane potential or reduced cell size, are more likely to acquire Tm cell features (7, 57–61).

When infection has been resolved (or cancer cells eliminated) and T cells undergo contraction, the anabolic processes that characterize effector T cell response fade (7, 62). During this phase, waning of antigen stimulation and inflammatory signals leads to the activation of AMPK and the MAPK-dependent inhibition of mTOR to activate autophagy and allow the generation and survival of Tm cells (6, 63–65). T cells that are able to engage catabolic processes to fuel OXPHOS will persist as Tm cells (57, 66), and the higher mitochondrial reserve capacity in these cells represents a bioenergetic advantage that underlies their rapid recall properties (67, 68). Distinct from Teff cells, Tm cells are characterized by elongated mitochondria with tight cristae structure (Figure 1C) (29), which together support efficient OXPHOS (19, 69). In line with this observation, T cells lacking the master regulator of inner mitochondrial membrane fusion OPA1 or the phosphatase PTPMT1, responsible for the rate-limiting step in cardiolipin synthesis, fail to develop into Tm cells in vivo (29, 70). Conversely, promotion of mitochondrial elongation, increasing of cardiolipin content, and inhibition of the repressor of OXPHOS efficiency MCJ1 are all strategies able to increase long-term survival and function of Tm cells (29, 70, 71).

## Metabolism of different Tm cell subsets

CD8^+^ Tm cells can be categorized broadly into three major subsets according to their functional properties, selectin molecule expression, and homing: central memory T (Tcm) cells, tissue-resident memory T (Trm) cells, and effector memory T (Tem) cells (72). Tcm cells continuously circulate through secondary lymphoid organs, while Trm cells are permanently located in peripheral tissues, where, with their rapid cytotoxicity, they are the first line of defense upon infection, but tend to be shorter-lived than Tcm cells. The third subset, Tem cells, are a heterogeneous population of T cells able to home to peripheral tissues that retain higher expression of effector molecules and support Trm cells in tissue protection by quickly migrating to the site of infection. Tcm cells largely downregulate effector properties during differentiation. Nevertheless, they are able to rapidly recall their function, produce a broader spectrum of cytokines, and undergo a more robust proliferation than Tem or Trm cells upon challenge from a previously encountered antigen. Although higher reliance on OXPHOS is a common metabolic trait of Tm cells, different Tm cell subpopulations have nuanced differences in terms of OXPHOS/glycolysis ratios as well as substrate utilization. Tem cells rely less on OXPHOS than Tcm or Trm cells. Indeed, Tm cells develop even when glycolysis is genetically enforced in T cells by deletion of the von Hippel Lindau (VHL) protein, but they are skewed toward a Tem phenotype (73). Tcm and Trm cells, although similar in terms of OXPHOS dependency, differ in terms of substrate utilization, with Trm cells having a unique requirement for acquisition of exogenous fatty acids to fuel mitochondrial respiration (74).

## Different substrates fuel Tm cells

**Figure 2:**
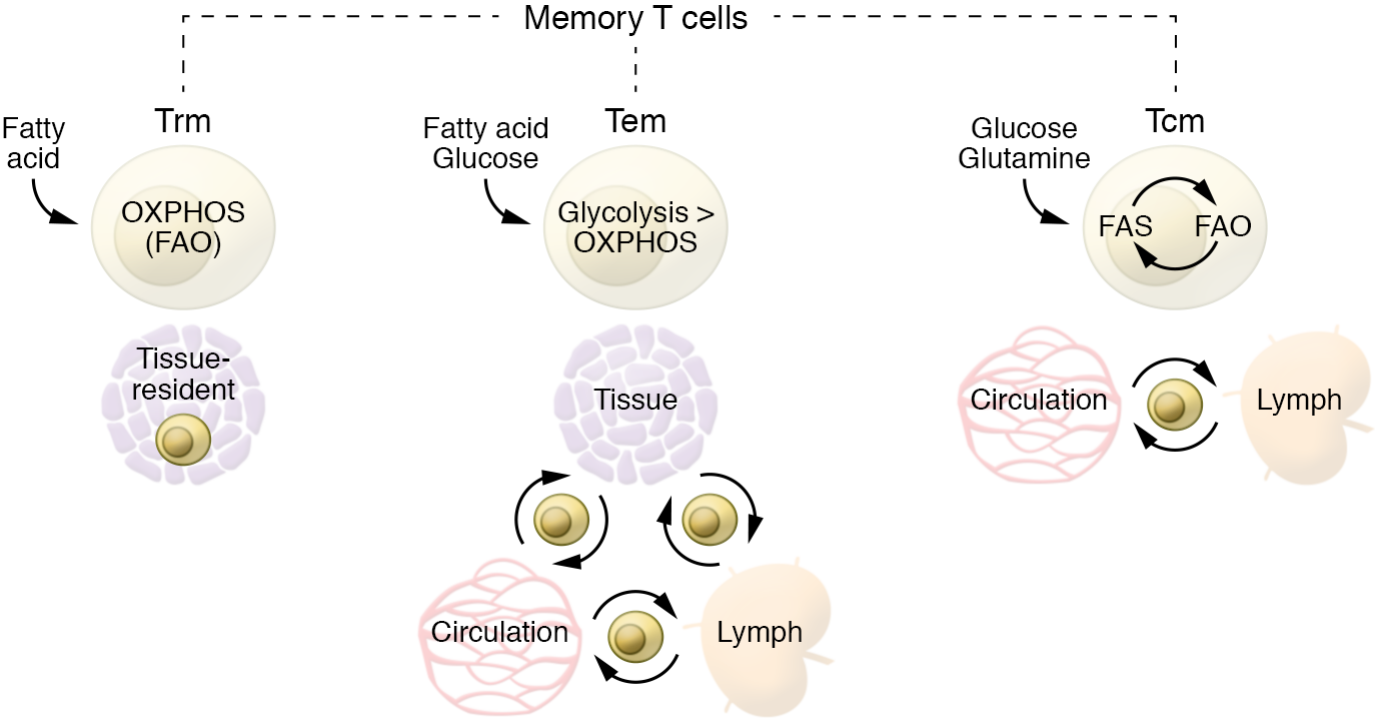
Metabolic features of different memory T cell subsets. Different Tm cell subsets preferentially use different substrates. Although they mainly rely on FAO for their energy demands, Tcm and Trm cells differ in the substrate of choice. Ex vivo Tcm cells and in vitro–generated IL-15–cultured Tcm cells engage an apparently futile cycle with the uptake of glucose used to generate fatty acids that are subsequently burned by FAO. These Tcm cells take up a lower amount of fatty acid compared with Teff or Trm cells and can even survive in a lipid-depleted medium (75). Conversely, Trm cells are able to acquire a greater amount of fatty acids directly from the microenvironment. Tem cells are relatively more metabolically active, as they are able to use multiple substrates to fuel glycolysis and OXPHOS.

Glucose, glutamine, and long-chain and short-chain fatty acids can all be acquired by Tm cells to fuel OXPHOS (75, 76). Nevertheless, different Tm subsets preferentially use different substrates (Figure 2) (77). Indeed, although they mainly rely on FAO for their energy demands, Tcm and Trm cells differ in the substrate of choice (57, 74). Ex vivo Tcm cells and in vitro–generated IL-15–cultured Tcm cells engage an apparently futile cycle with the uptake of glucose used to generate fatty acids that are subsequently burned by FAO (75). These Tcm cells take up a lower amount of fatty acid compared with Teff or Trm cells and even survive in a lipid-depleted medium (74, 75). Conversely, Trm cells are able to acquire a greater amount of fatty acids directly from the microenvironment (74, 78). In line with this observation, lipid chaperones like FABP4/5 and CD36 are specifically upregulated in Trm cells, and ex vivo exogenous supplementation of fatty acids increased spare respiratory capacity only in Trm cells, not in Tcm cells (74). A recent study challenged the central role for FAO in Tm cells (79). However, multiple explanations might reconcile these and previous findings, including thymic selection compensation, metabolic adaptation to alternative fuel sources, the increased utilization of short-chain over long-chain fatty acids, or compensatory enhanced peroxisomal FAO (23). Catabolic and anabolic processes coexist in Tm cells. They have indeed been observed not only for fatty acid synthesis and FAO (75), but also for gluconeogenesis and glycogenolysis (76), and for triacylglycerol synthesis and lipolysis (80). When the metabolic equilibrium between these pathways is genetically or pharmacologically perturbed, Tm cells fail to develop. These observations highlight the metabolic flexibility of Tm cells. Nevertheless, additional studies are required to further investigate and clarify the role and crosstalk of multiple anabolic and catabolic processes in these cells.

Multiple observations show that pharmacologic or genetic interference with mTOR activity promotes Tm cell generation (6, 7). mTOR is a critical hub for sensing amino acid content and the metabolic status of the cell. It is therefore not surprising that amino acid availability, transporter expression, and amino acid uptake are all critical for T cell activation and expansion, naturally limiting Tm cell development (81, 82). Glutamine uptake via ASCT2 coregulates leucine transport and mTOR upon T cell activation, and genetic ablation of *Slc1a5* (the gene encoding ASCT2) results in accumulation of CD4^+^ Tem cells with no apparent differences in CD8^+^ cell subsets (83, 84). In a different setting, limiting serine availability during CD8^+^ T cell activation and primary expansion impairs proliferation upon secondary infection, resulting in limited bacterial clearance (85). Genetic inhibition of one-carbon metabolism — fed by serine — also impairs CD4^+^ T cell activation by blocking mitochondrial biogenesis (86). These observations point to a specific role for amino acid availability and utilization during different phases of T cell development. More nuanced approaches including inducible knockout mouse models in the context of infection (with Cre recombinase activated only at the peak or after the effector phase) are required to dissect how specific amino acid requirements might impact Tm over Teff cell generation and function. Notably, in addition to fueling metabolic programs, amino acids also bridge metabolism and epigenetics. Methionine availability, for example, regulates the biosynthesis of the universal methyl donor *S*-adenosyl-l-methionine (SAM) and the H3K4 methylation (H3K4me3) state of CD4^+^ T cells, controlling proliferation and cytokine production of Th17 cells (87).

## Epigenetic control of metabolism in Tm cells

Specific signaling, metabolic, or antigen-driven information is imprinted during the primary effector phase and conserved over time, contributing to immune memory through epigenetic modification of histones (9). Interestingly, in addition to directly modulating bioenergetic pathways via substrate availability, metabolites can act as signaling molecules, often by modifying the epigenetic landscape of immune cells (88, 89). High levels of acetate experienced by T cells at the peak of effector phase are responsible for the acetylation of GAPDH, stimulating higher glycolysis, while also acetylating histone 3 lysine 9 (H3K9), thus enhancing chromatin accessibility of specific promoter regions of Teff cell–associated genes in Tm cells, favoring their rapid expression upon restimulation (90–92). Another metabolite that influences the epigenome is α-ketoglutarate, which reduces the expression of the DNA methyltransferases *Dnmt3a* and *Dnmt3b* via inhibition of the transcription factor OTX2 (93). DNMT3A-mediated erasure of de novo methylated regions during the Teff cell phase regulates re-expression of Tn cell–associated genes, allowing Tm cells to differentiate from a fate-permissive subset of Teff cells (94); this explains the partially conserved DNA methylation profile between Teff and Tm cells in both mice and humans and suggests a lineage relationship between these two populations (94, 95). There are many more examples of how metabolism influences the epigenome, and a more comprehensive overview of the epigenetic control of T cell fate and differentiation can be found in other recent reviews (9, 96).

## T cell dysfunctions in patients with mitochondrial diseases

Mitochondrial diseases (MDs) are the most common group of inherited metabolic disorders and arise from mutations in mitochondrial genes encoded by the nuclear (nDNA) or mitochondrial (mtDNA) genome (97, 98). They are heterogeneous in etiology (mutations in nDNA or mtDNA) and in inheritance mechanisms (maternal for mtDNA mutations, autosomal dominant/recessive or X-linked for nDNA mutations) (97, 98). Moreover, although all MDs are characterized by dysfunctional OXPHOS, mtDNA integrity, or mitochondrial maintenance, MDs manifest in a phenotypically diverse spectrum often affecting muscle, heart, or brain physiology with variable penetrance and severity (97, 98). Recent advances in the immune characterization of patients affected by (as well as mouse models of) MDs suggest that immune dysfunction might be added to the features of MDs (99). Up to half of patients with MD experience recurrent or severe upper respiratory tract infections, often resulting in life-threatening conditions (100, 101). This percentage increases to almost 90% of pediatric MD patients (101), with sepsis (55%) and pneumonia (29%) as the two most common causes of death (102). Immune dysfunction in MDs might be due to multiple factors, including the higher incidence of leukopenia observed in patients affected by Barth syndrome, Pearson syndrome, and Leigh syndrome (103). Defects have manifested in lower Tm (CD45RO^+^) cell frequencies in a pediatric cohort of MD patients (101), deficient cytokine production in a small cohort of Barth syndrome patients (70), or impaired antibody production upon vaccination, which was observed in a case of fatal neonatal-onset mitochondrial respiratory chain disease (104). Interestingly, supporting a role for FAO and CPT1a in shaping an efficient long-lasting immune response, a study of Native Alaskan children carrying a hypomorphic variant of *CPT1a* showed a higher incidence of respiratory tract infection and otitis in comparison with the control group (105).

More broadly speaking, the immune phenotype in patients with MDs could result either from functional defects intrinsic to T cells (or other immune cells) or from cell-extrinsic mechanisms (Table 1). Febrile temperature, a physiologic response to infection, increases basal metabolic rate (10% higher per 1°C increase in body temperature) (106). In patients with MDs the increase in metabolic rate during an infection coupled with the impairment of OXPHOS might exacerbate lactic acidosis, an already common feature of MDs (107) that is known to inhibit T cell function (108–110). Genetic defects in OXPHOS might also directly impact Tm cell development given the established role of mitochondrial respiration during this phase (29, 57, 66, 70), potentially contributing to the impaired immune memory and partially explaining the repetitive susceptibility to bacterial and viral infections of patients with MDs.

Thus, considering that infections can be more deleterious in children with inborn errors of metabolism (IEM), preventing infections via vaccination is key. Nevertheless, the same mechanisms that negatively affect the immune response to natural infections might do the same in response to vaccines. Despite this reasonable concern, multiple studies reported positively on the immunogenicity, safety, and tolerability of vaccines in children with IEM (111, 112). Vaccination regimens are recommended in IEM patients and are not associated with increased risk of serious adverse effects during the month after vaccination, although the risk might be more pronounced for the more metabolically unstable patients (113). Administration of live attenuated vaccines should be evaluated carefully in immunocompromised patients (111). While these considerations are valid and vaccines are highly recommended for patients with MDs, further MD-specific studies are needed to establish pathology-specific guidelines and vaccine regimens — perhaps by adding additional boost doses at regular intervals, as is often recommended for immunocompromised patients.

Notably, although not of genetic etiology, the progressive decline in mitochondrial function reported during aging (114, 115) also involves T cells (86, 116, 117), and could be one factor contributing to lower T cell responses to vaccines in elderly populations (118).

## Impact of metabolic health on T cell function

Obesity and metabolic syndrome are major public health issues, with numbers of obese people increasing worldwide (119). Metabolic disruptions leading to metabolic syndrome include the combination of at least three of the following factors: central adiposity, elevated blood glucose and plasma triacylglycerols, high blood pressure, and low plasma HDL-cholesterol (120). Moreover, metabolic syndrome is often characterized by endothelial cell dysfunction, atherogenic dyslipidemia, insulin resistance, and chronic low-grade inflammation (121). Metabolic alterations and inflammation engage in a vicious cycle with T cell activation, senescence, and proinflammatory cytokine production that worsens pathologic conditions and results in higher rates of vaccine failure and complications from infection (122, 123). Mechanistically, in addition to its effects on innate immune cells, leptin — the levels of which are increased in obese or metabolically impaired individuals — also modulates adaptive immunity by inducing expression of activation markers on T cells (124), inhibiting proliferation of Tm cells (125, 126), and polarizing Th cells toward Th1 proinflammatory phenotype while simultaneously inhibiting Treg function (127–129). Hyperinsulinemia as an adaptation to systemic insulin resistance is a common feature of obesity, fostering type 2 diabetes onset and progression. Multiple studies showed that the insulin receptor (INSR) is also present on T cells, where it modulates glucose and amino acid uptake and is generally upregulated during T cell activation (130, 131). Whole-body knockout of INSR showed reduced cytokine production, proliferation, and migration, as well as increased apoptosis of T cells, although the results from this model were confounded by the underlying hyperglycemia associated with systemic loss of INSR function (132). T cell–intrinsic defects were confirmed by selective ablation of INSR on T cells and observation of deficiencies in proliferation and cytokine production that resulted in impaired inflammatory and T cell–specific responses to influenza virus (133). These observations could well contribute to the higher susceptibility to severe infections and cancer as well as the weakened vaccine effectiveness associated with systemic insulin resistance observed in obese people (134–137).

## Overcoming metabolic competition in the tumor microenvironment to improve Tm cells

Although T cell therapy has shown great preclinical and clinical success in treatment of hematologic malignancies (138, 139), its efficacy in the treatment of solid tumors has been disappointingly low (140). Many studies have combined T cell therapy with administration of proinflammatory cytokines or checkpoint blockade inhibitors to improve success (141), but the severe side effects as well as the unsatisfactory results observed prove the necessity of developing new approaches (142–144). Lack of antigen recognition, chronic activation and exhaustion, and hyporesponsiveness of T cells are common mechanisms of immune evasion in cancer (145–147). In recent years, metabolic competition in the tumor microenvironment (TME) has been increasingly recognized as an additional effective immune escape strategy (148). Many cancer cells rely on glucose through Warburg metabolism and compete with T cells for this substrate, leading to lower concentration of glucose in the TME compared with plasma (149–151). The parallel use of mouse models of regressing and progressive sarcoma tumors and melanoma-bearing Braf/Pten mice revealed that the TME of progressive tumors had a lower glucose concentration compared with that of regressive ones (149, 150). These two studies together formally cemented the idea that nutrient competition occurs in the TME, and that this as a distinct mechanism can drive cancer progression. A similar experiment comparing TME of implanted tumors from two pancreatic cell lines derived from early- and late-stage pancreatic ductal adenocarcinoma (PDAC) showed lower glucose in the interstitial fluid of advanced PDAC tumors (152). A recent study analyzing human renal cell carcinoma and mouse subcutaneous MC38 tumors compared with adjacent healthy tissue challenged the idea of glucose restriction in the TME (153). Moreover, glucose uptake measured in vivo by ^18^F-fluorodeoxyglucose PET imaging revealed that T cells are able to acquire glucose in the TME, although they remain functionally impaired. Overall this study suggested that there is selective nutrient partitioning among different cells in the TME. Inhibiting the higher glutamine uptake in cancer cells unleashed glucose uptake by TME-resident cells, including T cells, beyond basal levels, restoring their function; this suggests that glutamine metabolism suppresses glucose uptake by T cells without glucose being a limiting factor in the TME (153). In a different study, divergent metabolic programs upon glutamine metabolism blockade were also observed between cancer cells and T cells, and they were associated with increased glucose availability in the tumor and functional and metabolic rescue of T cells (154). It has also been shown that checkpoint blockade therapy (149) or inhibition of the N6-methyladenosine RNA demethylase FTO (155) can directly impact tumor cell metabolism while increasing glucose uptake by T cells. While the presence and extent of glucose restriction in TME might reflect cancer type heterogeneity, competition for metabolites between cancer and immune cells remains a key factor governing the balance between cancer progression and regression.

**Figure 3:**
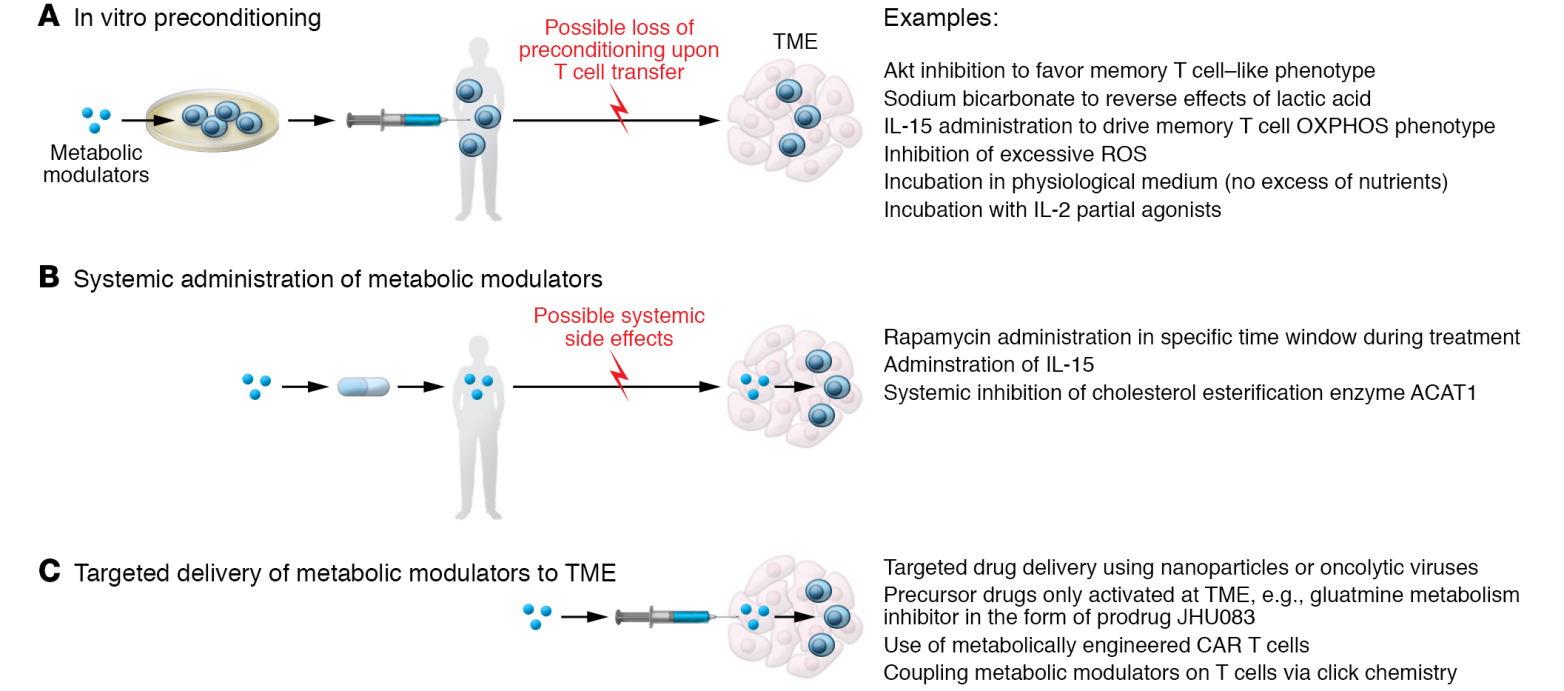
Metabolic interventions in cancer immunotherapy. Three main scenarios for metabolic interventions in the context of cancer immunotherapy can be imagined: (**A**) In vitro preconditioning to prime T cell metabolism before autologous in vivo transfer. One caveat to consider in this approach is the loss of the induced preconditioning upon T cell transfer in vivo. (**B**) Systemic administration of drugs that alter tumor and T cell metabolism. A limitation of this strategy is the potential for the development of systemic side effects. (**C**) Targeted delivery of metabolic modulators directly in the TME or administration of precursor drugs selectively activated in the TME could potentially overcome the issues described for the first two approaches.

Notably, many interventions to overcome TME inhibitory effects on T cells coincide with treatments that either directly stimulate mitochondrial activity or mimic pro-memory metabolic features. With regard to harnessing metabolism for therapeutic interventions in cancer immunotherapy, three main scenarios have been envisioned: in vitro metabolic preconditioning, systemic in vivo metabolic treatments, and targeted delivery of metabolic modulators in the TME (156); and multiple strategies have been designed to address them (157) (summarized in Figure 3). The first approach embraces in vitro preconditioning of cells with metabolic modulators before adoptive transfer. Examples include inhibiting Akt function to favor a more Tm cell–like metabolic phenotype in mouse melanoma models (59); using sodium bicarbonate to reverse the lactic acid–induced interference with T cell glycolysis and cytotoxic function in mouse models and patients with acute myeloid leukemia (110); and transiently exposing donor lymphocytes to 39°C prior to infusion in a myeloid leukemia mouse model (158). Similarly, preconditioning T cells with IL-15, which drives Tm cell differentiation and metabolically increases spare respiratory capacity (66), has similar positive results in HER2-positive tumors, leukemia, and glioma models (159–161). An approach based on the transient rest of CAR T cell receptor signaling has been suggested to restore functionality of T cells and reverse their exhausted phenotype (162). Interestingly, from a metabolic perspective, continuous T cell stimulation in a hypoxic microenvironment drives T cell exhaustion by inducing mitochondrial stress (163). Pharmacologic treatments aimed at reducing reactive oxygen species (ROS) or lowering tumor hypoxia improve response to immunotherapy in mice (163). Therefore, a rest period could also reinvigorate mitochondrial metabolism to sustain long-term T cell persistence and function. It should also be considered that in vitro generation and amplification of tumor-infiltrating lymphocytes for adoptive cell transfer or CAR T cells often require incubation in supraphysiologic concentrations of nutrients such as glucose, or cytokines like IL-2 (164). Based on preclinical observations (7, 30, 165), a transient preincubation in a more physiologic medium inducing glucose restriction or treatment with rapamycin (or analogs) is a promising strategy to prime T cells for Tm cell differentiation (161, 164, 166, 167). Along the same line, the treatment of CD8^+^ T cells with the engineered IL-2 partial agonist H9T improves mitochondrial fitness and promotes a stem cell–like state (168). Boosting mitochondrial elongation or cardiolipin content and blocking the repressor of OXPHOS efficiency MCJ1 are all metabolic preconditioning strategies to increase long-term survival and function of Tm cells in mouse models (29, 70, 71).

This in vitro metabolic preconditioning strategy has the clear caveat that it might be reversed or lost when cells are transferred in vivo and approach the TME. To overcome this issue, a second strategy could employ systemic administration of metabolic modulators. Various clinical trials are under way to test clinical efficacy of vaccination with NY-ESO-1 tumor antigen–based vaccines in combination with rapamycin treatment (ClinicalTrials.gov NCT01536054, NCT02833506, and NCT01522820) or IL-15 superagonists (NCT02384954) (169). Preclinical studies in mouse models showed that systemic inhibition of the cholesterol esterification enzyme ACAT1 potentiates the CD8^+^ T cell antitumor response by increasing cholesterol concentration in the plasma membrane and enhancing T cell receptor clustering and signaling (170). Possible systemic side effects and complexity of pharmacokinetics of the compounds used are a persistent issue with these nevertheless promising approaches.

A third strategy is targeted delivery of metabolic modulators directly in the TME. This strategy includes drug delivery using nanoparticles (171), oncolytic viruses (172), use of metabolically engineered CAR T cells (173, 174), or precursor drugs selectively activated in the TME (154). In line with this idea, an intriguing strategy based on click chemistry (175) has been used to backpack the ACAT1 inhibitor avasimibe directly on T cell membrane to locally increase cholesterol, improving T cell receptor clustering and, thus, T cell activation and function in mouse models of glioblastoma and melanoma (176). A similar approach could be envisaged coupling other metabolic modulators directly on T cells, creating a new generation of combinatorial anticancer therapies. Additional strategies exploit other features of the TME to achieve a site-specific activation of T cells or drugs. Stemming from the observation that CAR T cell efficiency can be manipulated by metabolic engineering of these cells (e.g., via CD28 or 4-1BB) (36–40), new improved versions of these cells have been generated. Oxygen-sensing CAR T cells activated by the hypoxic TME have been developed to reduce possible off-target effects in solid tumors and have proven effective in mouse models of hypoxic HN3 tumors (174). Alternatively, precursor drugs can also be engineered to become active only in the tumor. For example, to avoid systemic toxicity of comprehensive glutamine metabolism inhibitors, precursor drugs have been designed to be cleaved by specific proteases at the tumor site, where they can locally exert their inhibitory function and promote OXPHOS and Tm cell development (154).

An emerging area of research aims at investigating how systemic metabolic interventions like glucose restriction or ketogenic diet might be exploited to boost anticancer treatment. This research area stems from pioneering work showing that the systemic hyperinsulinemia observed in cancer patients treated with a PI3K inhibitor, which is able to reactivate mTOR signaling in cancer cells, can be blocked by a ketogenic diet, allowing more effective control of tumor growth than PI3K inhibitor treatment alone (177). Despite this promising observation, ketogenic diet interventions may only be valid in PI3K-dependent tumors, and could be detrimental in other tumors that are able to metabolically adapt their growth to the use of ketone bodies as a fuel source.

## Developing vaccines that elicit efficient T cell responses

A persistent challenge in vaccine development is the generation of vaccines able to elicit efficient T cell responses and Tm cell generation in addition to humoral responses. This could be particularly important for patients with B cell deficiency or functional decline. Many modern vaccines, which are often not based on live attenuated pathogens, are not highly effective at priming persistent T cell immunity (4). This might be related to the fact that, unlike B cells, T cells recognize antigen in the context of MHC on APCs, which requires cross-presentation of antigen by APCs, higher amounts of the initial antigen dose, and a longer persistence of antigen. Vaccines that efficiently prime a T cell response are often live attenuated vaccines, like those used against yellow fever (YF-17D) or smallpox (178, 179). Conversely, vaccines against influenza virus poorly activate T cells (180). In order to improve vaccine-mediated T cell responses, multiple strategies have been deployed, including the use of various DNA-based vaccines and viral vectors, the study of specific prime-boost regimens, and adjuvant combinations. Often, these strategies showed limited success (181–183). The mRNA vaccines BNT162b1 and mRNA-1273, developed during the COVID-19 pandemic, were shown to promote high frequencies and persistence of SARS-CoV-2 receptor-binding domain–specific CD4^+^ and CD8^+^ T cells highly effective in IFN-γ production (184–186). The mechanisms, which remain elusive, might be related to how mRNA is expressed by target cells, and to the persistence of antigen expression. Adenovirus (Ad) vector–based vaccines using the backbone of human Ads (huAd5 or huAd26) or chimeric vectors based on chimpanzee viruses (ChAdOX1) are also able to elicit a CD8^+^ T cell response to viral antigens (187–189) owing to their ability to promote a niche of persistent antigen presentation in fibroblastic stromal cells in the lungs (190).

While new vaccines are developed, another line of research includes exploiting metabolic features of innate and adaptive immune cells to generate more efficient T cell activation during vaccination using metabolic adjuvants targeting the activity of mTOR or the amino acid sensor GCN2 (191). Alternatively, in line with their role to protect tissue immediately upon infection, the ability of adjuvants and vaccines to specifically induce antigen-specific Trm cells has also been intensively studied (192). In this regard, it is of great interest to integrate metabolic phenotyping into vaccinology (193, 194). Changes in plasma metabolites upon vaccination have been investigated in pioneering studies on the live attenuated shingles vaccine Zostavax, showing how sterol metabolism integrates cellular and humoral responses (195). In this study alterations in plasma metabolites were observed already at day 1 after vaccination and anticipated the concordant transcriptional changes observed 48 hours later, suggesting that changes in metabolism precede and maybe instruct changes at the transcriptional level (195). It could therefore be envisioned that the implementation of similar systems biology methods, fed also by metabolomics data, could be used to identify more promising vaccine candidates earlier in the developmental stage, or to identify vaccine nonresponders in particularly fragile populations and accordingly modulate other pharmacologic and nonpharmacologic interventions.

## Concluding remarks

The current challenge of the COVID-19 pandemic has made clear that new creative ways to confront long-lasting immunology questions are key for advancing therapies. Unveiling the metabolic circuits regulating immunity and combining discoveries from cancer metabolism, vaccine development, and T cell biology are central strategies to tackle this and future pandemics as well as improving our current treatment of cancer, infections, and autoimmune diseases. The subclinical presence of mitochondrial diseases and metabolic disorders could be a critical factor to take into consideration when prognosis and therapies for cancer, infections, and autoimmune diseases are clinically discussed or vaccine efficacy is evaluated.

## Figures and Tables

**Table 1:**
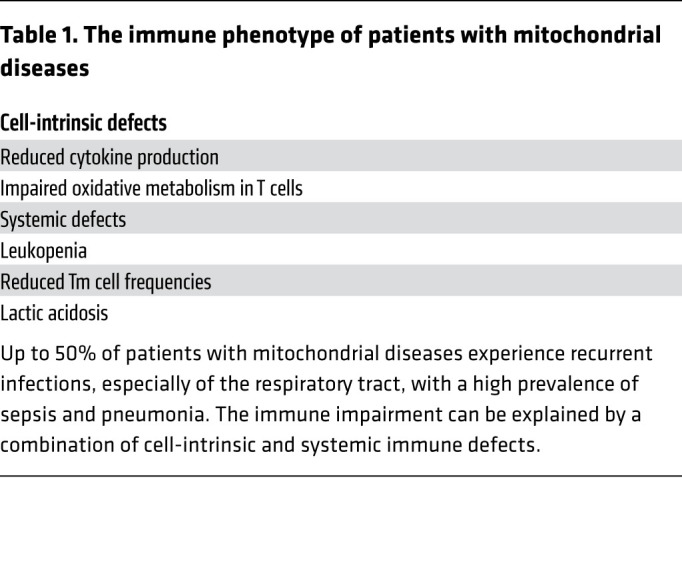
The immune phenotype of patients with mitochondrial diseases

## Version 1 01/04/2022

Electronic publication
